# Supplementary material for: Delayed Antarctic sea-ice decline in high-resolution climate change simulations
Source: Nat Commun. 2022 Feb 2;13:637. doi: 10.1038/s41467-022-28259-y (PMC8810850; doi:10.1038/s41467-022-28259-y)
Supplement: Supplementary file 1 — Supplementary Information [file 41467_2022_28259_MOESM1_ESM.pdf]

Supplementary Information for

Delayed Antarctic sea ice decline in high-resolution climate change simulations

Thomas Rackow, Sergey Danilov, Helge F. Goessling, Hartmut H. Hellmer, Dmitry V. Sein, Tido Semmler, Dmitry Sidorenko, and Thomas Jung

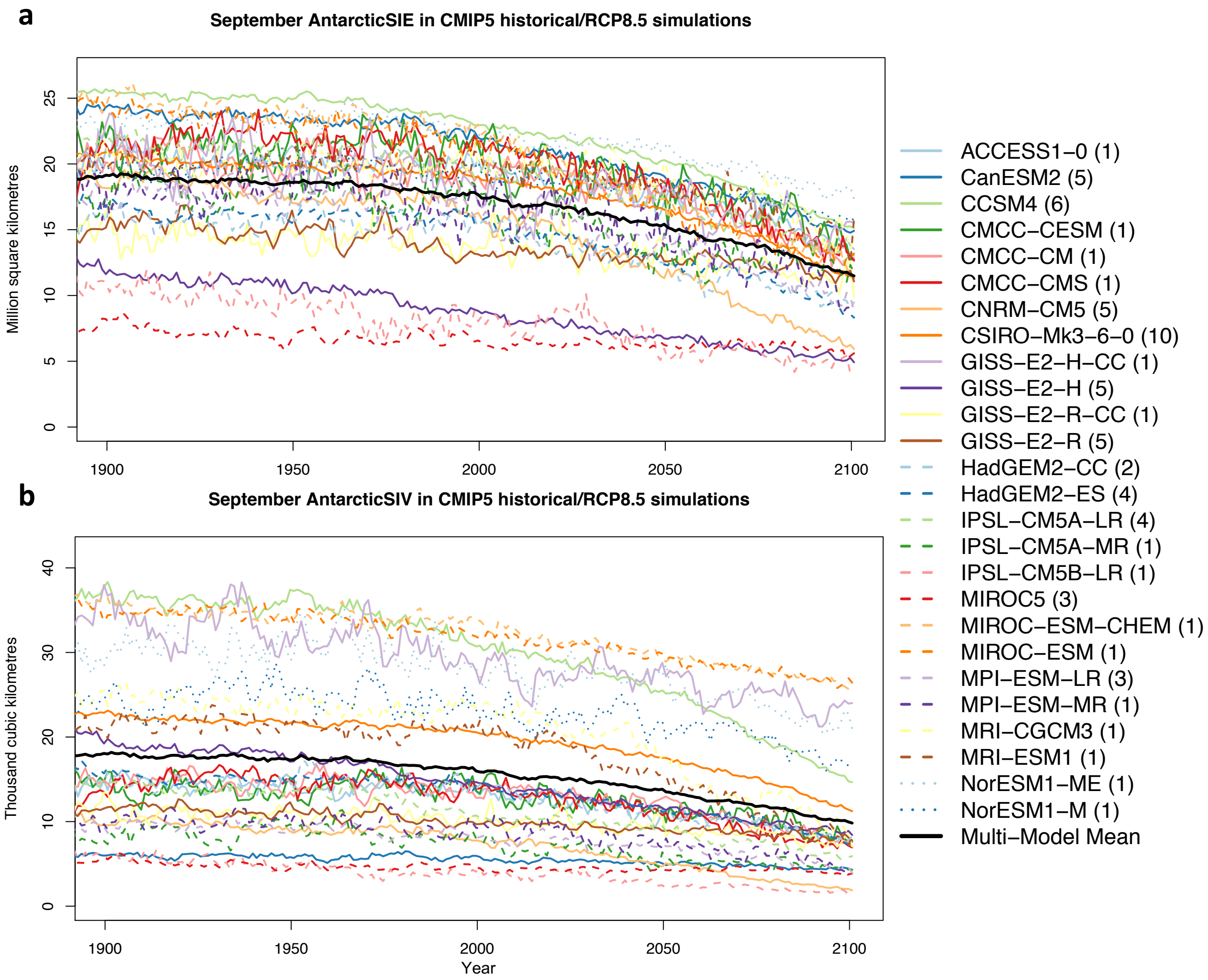

Supplementary Figure 1: Projected sea ice changes (historical + RCP8.5) in current climate models participating in the Coupled Model Intercomparison Project (CMIP5). a, Antarctic sea ice extent time series and **b,** time series of sea ice volume. For every model (see legend), the ensemble-mean is given, which is based on different numbers of ensemble members (numbers in brackets). The bold black line denotes the CMIP5 multi-model mean.

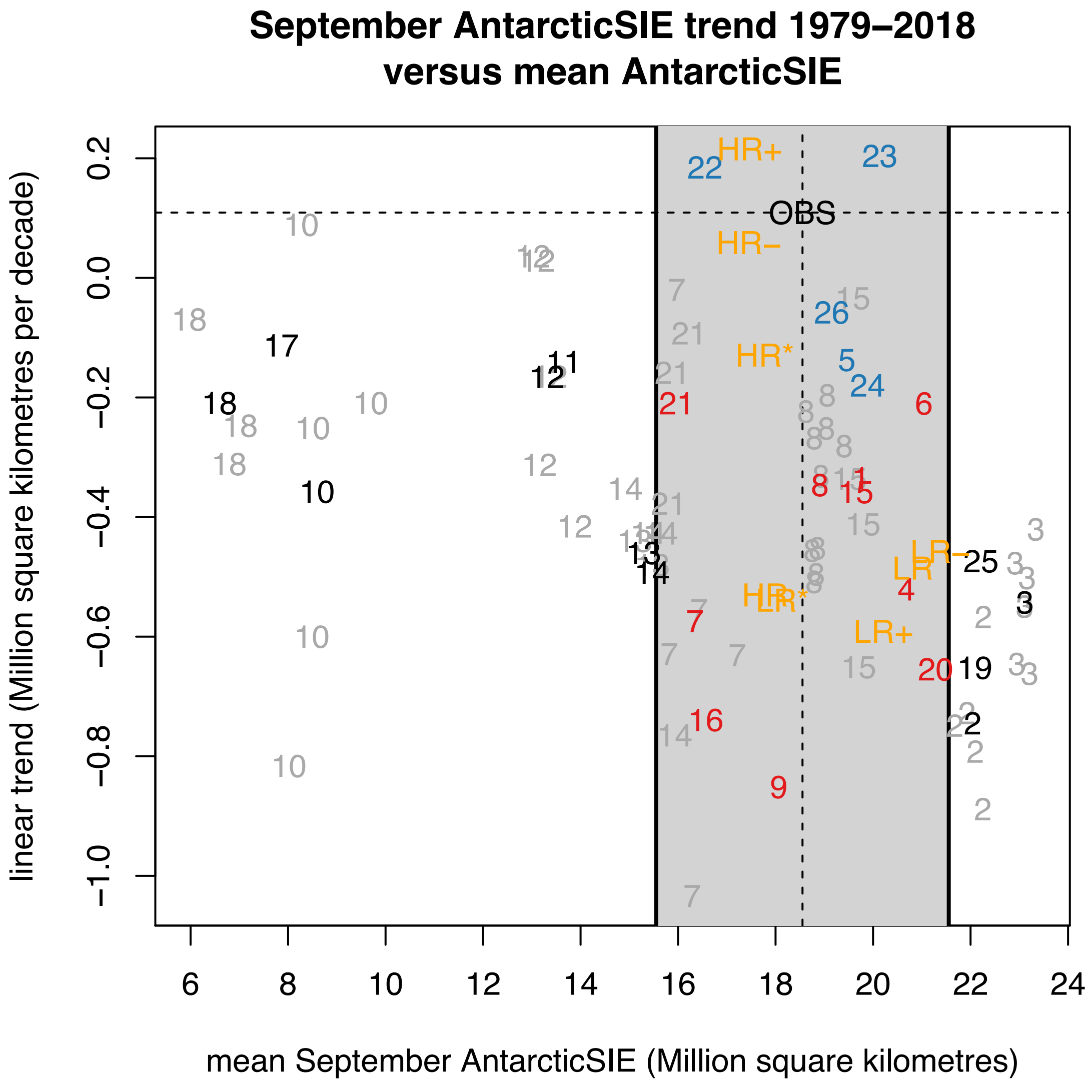

Supplementary Figure 2: Same as Fig.1a of the main text, but with all CMIP5 ensemble members added with grey symbols, and different AWI-CM results with yellow symbols. “LR” and “HR” denote AWI-CM results for the satellite period (1979—2018), and periods shifted by ±10 years are given as “LR+”/”HR+” (1989—2028) and “LR-“/”HR-“ (1969—2008). Additionally, “LR*”/”HR*” describes the later 40yr-period 2019-2058, which is unaffected by the HR model’s initial adjustment and therefore plotted in Fig.1.

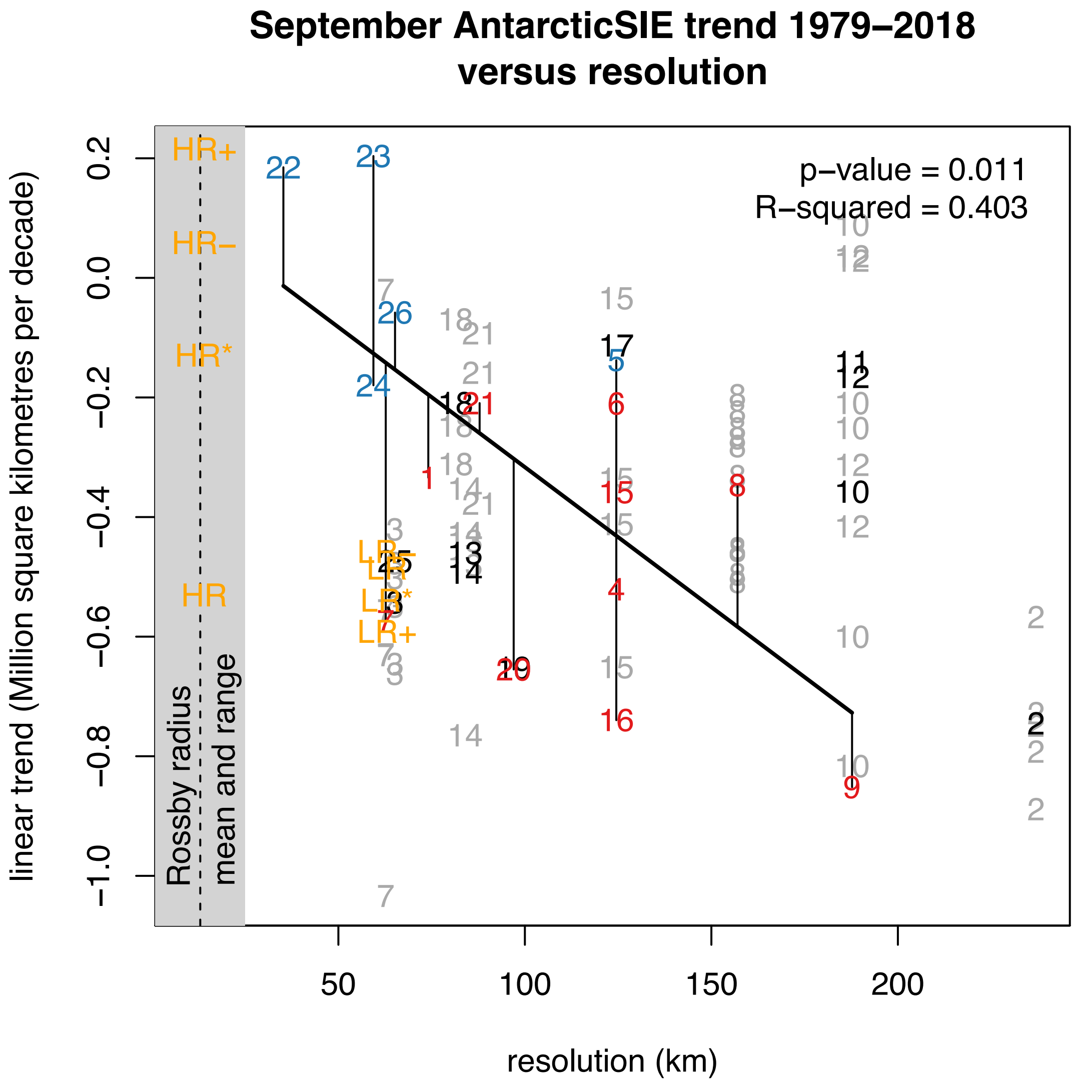

Supplementary Figure 3: Same as Fig.1b of the main text, but with all CMIP5 ensemble members added with grey symbols, and different AWI-CM results with yellow symbols. “LR” and “HR” denote AWI-CM results for the satellite period (1979—2018), and periods shifted by ±10 years are given as “LR+”/”HR+” (1989—2028) and “LR-“/”HR-“ (1969—2008). Additionally, “LR*”/”HR*” describes the later 40yr-period 2019-2058, which is unaffected by the HR model’s initial adjustment and therefore plotted in Fig.1.

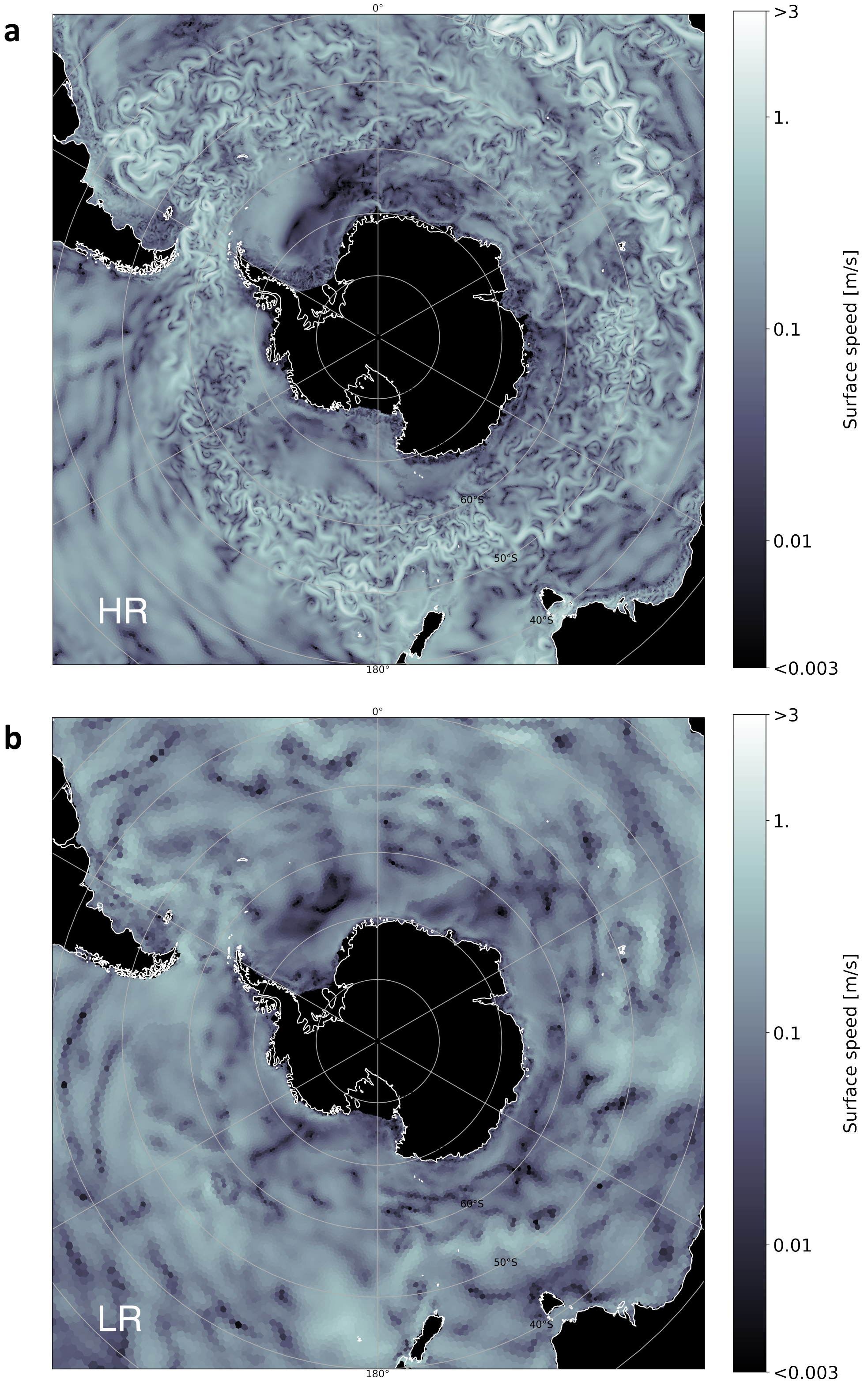

Supplementary Figure 4: Snapshots of the Southern Ocean surface circulation [m/s] for September 1990 in the two different AWI-CM ocean configurations. a, The high-resolution HR grid, **b**, the low-resolution LR grid. Note the logarithmic scale of the panels. The Antarctic Circumpolar Current is represented with eddy-permitting resolution or better in HR when compared to the local Rossby radius of deformation (see also Figure S5).

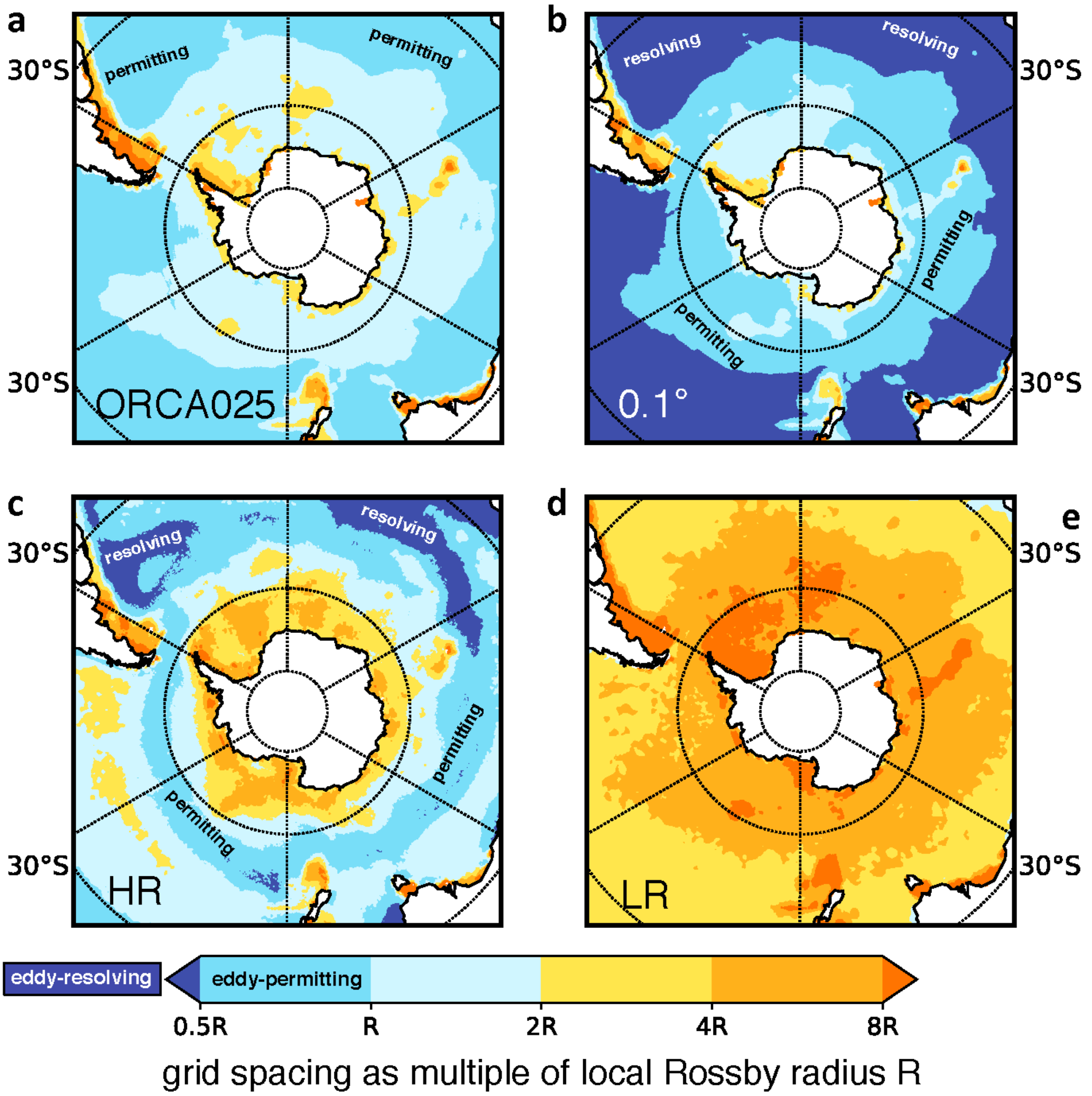

Supplementary Figure 5: Grid spacing of different ocean model grids as multiples of the local Rossby radius of deformation. a, ORCA025 NEMO (0.25°), **b**, a typical 0.1° ocean grid, **c**, AWI-CM-HR grid, and **d**, AWI-CM-LR.

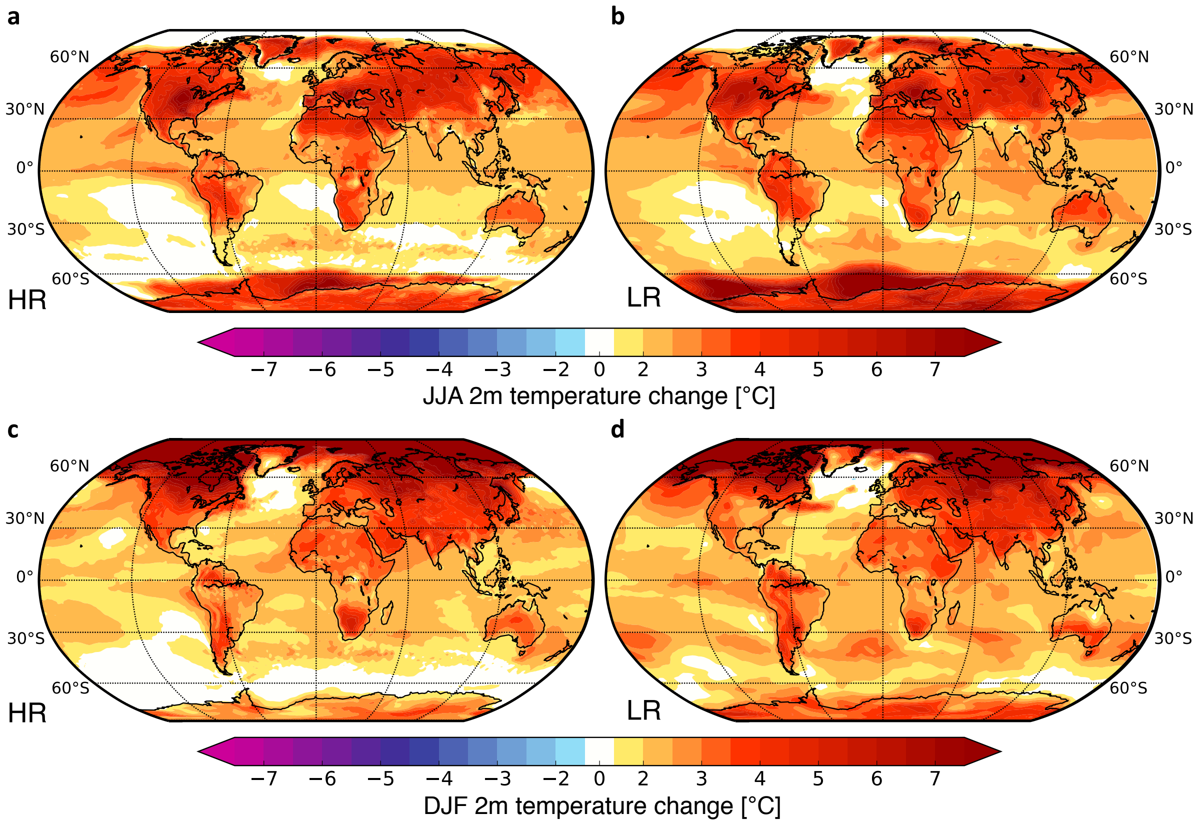

Supplementary Figure 6: Global view of projected 2m temperature changes for the end of the 21st century (2070–2099, relative to 1990–2019) in the JJA and DJF seasons. The projections for **a,c** HR and **b,d** LR are based on a representative high-emission concentration pathway (RCP8.5 scenario). The figure gives a global view for the changes reported on in Fig.2a,b and Fig.2d,e.

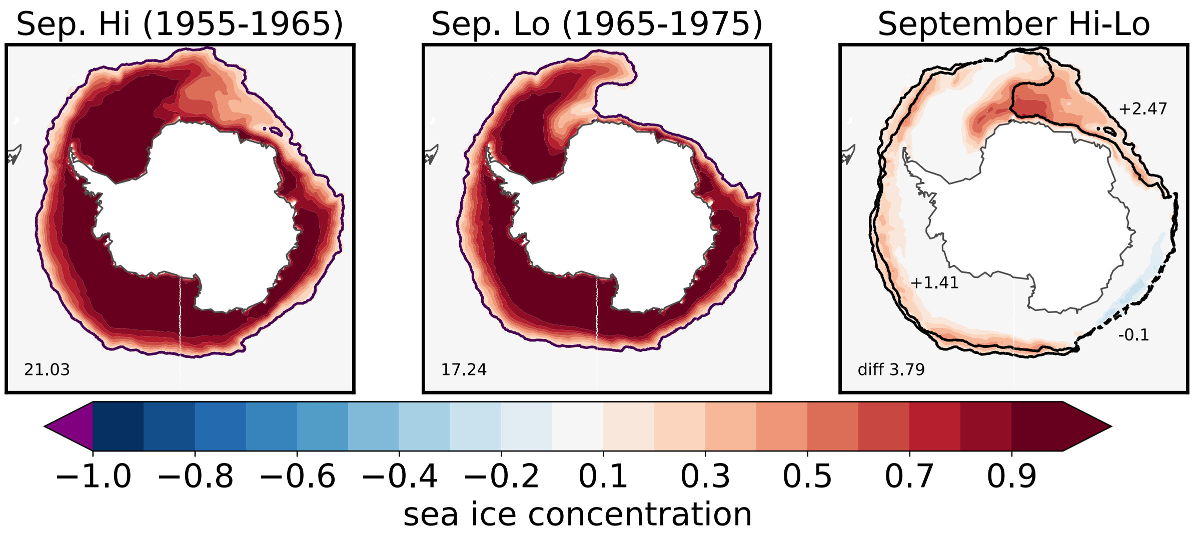

Supplementary Figure 7: Composites of September Antarctic sea ice concentration (in colors) and extent (black contours) for two periods of extraordinarily high (1955—1965, Hi) and low (1965—1975, Lo) sea ice extent in the HR simulation, immediately after the HighResMIP spinup period. The difference (Hi-Lo) is shown in the right panel. Solid lines outline areas where concentration changes translate to increased sea ice extent in Hi compared to Lo (+3.89 million km^2, dashed lines denote decreased extent (-0.1 million km^2). About 37% of the positive change occurs in a narrow band along the sea-ice edge in the Pacific and Western Weddell Sea sector, while 63% are from the Eastern Weddell Sea and Indian Ocean sector (annotated numbers in right panel).

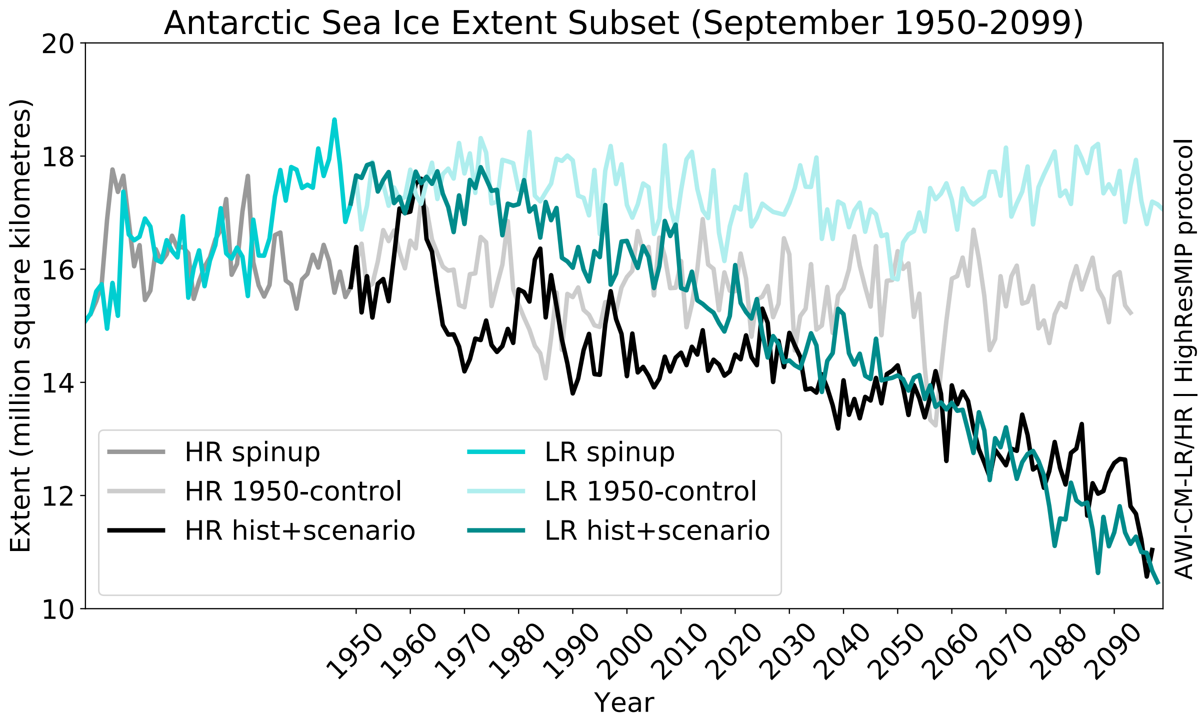

Supplementary Figure 8: Antarctic sea ice extent, without the region 0°—70°E, in high-resolution (HR; bold black line) and low-resolution (LR; bold dark green line) AWI-CM configurations. The 1950-control simulations are shown in lighter shades.

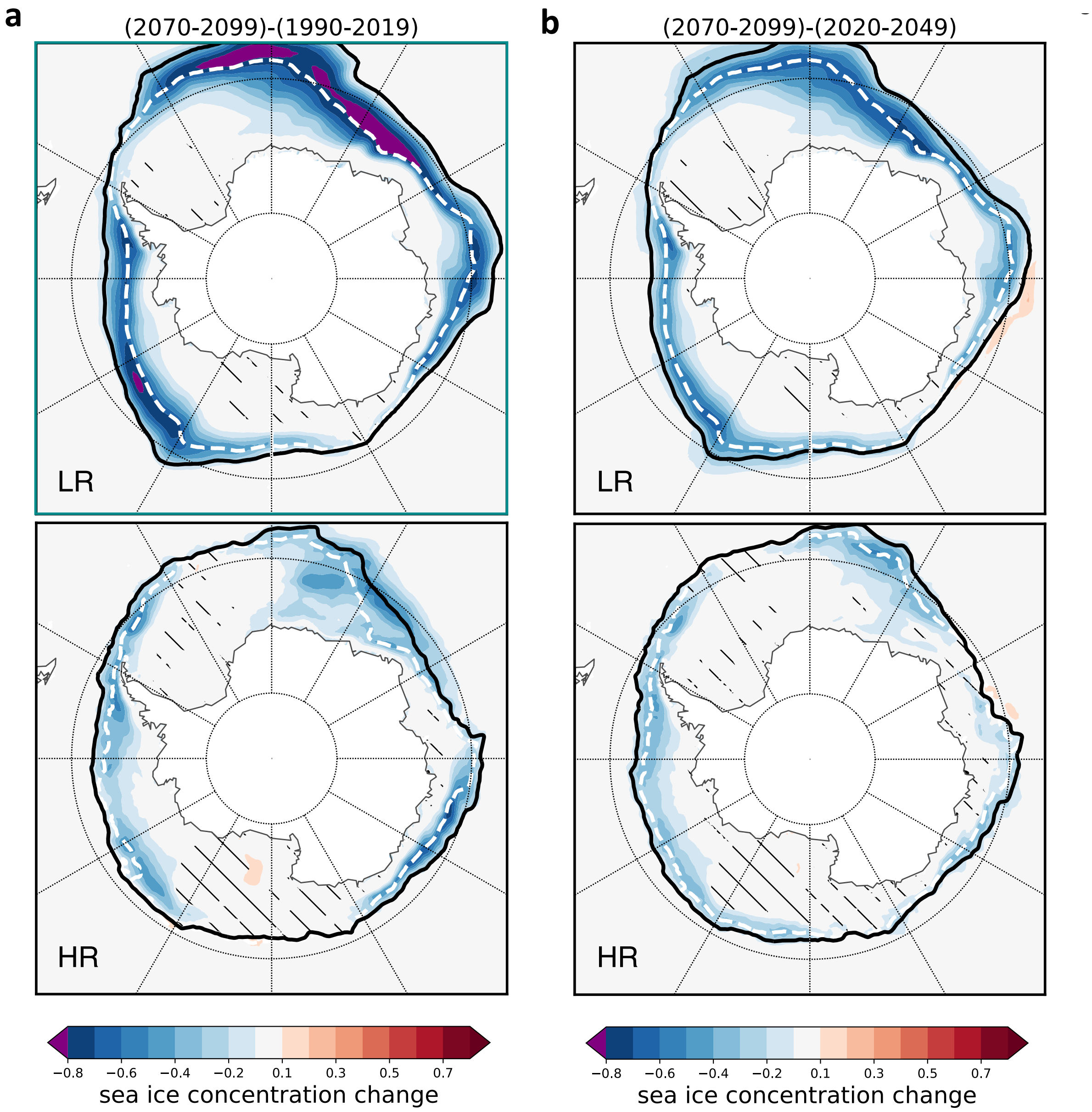

Supplementary Figure 9: Patterns of projected late 21st century (2070—2099) sea-ice concentration changes in September in LR (top row) and HR (bottom row). a, Projected changes with respect to 1990—2019, where the sea ice extent (and volume) in HR appears to be in a slight trough. **b,** Same as panel **(a)**, but for the later baseline 2020—2049, after the multi-decadal initial adjustment in HR. Black contours show the sea ice extent for the respective baseline period (1990—2019 or 2020—2049), white dashed contours are for 2070—2099. The "delta approach" (see Methods) has been applied to extract the climate change signals. The signals are significantly larger than typical unforced 30yr-variability (standard deviation) in the 1950 control simulations (see Figure S10). Hatching indicates non-significant regions where the concentration changes are smaller than two standard deviations.

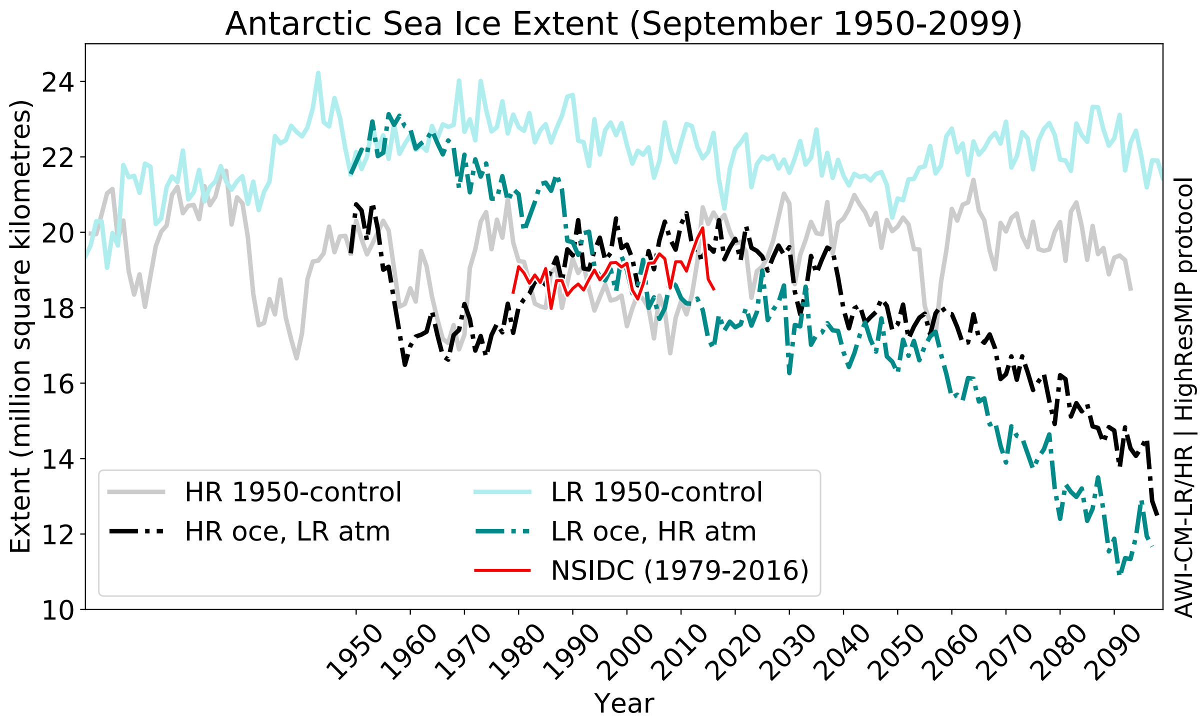

Supplementary Figure 10: Time series of Antarctic sea ice extent in mixed-resolution AWI-CM configurations with the HR ocean (black dashed; LR atmosphere above) and LR ocean (dark green dashed; HR atmosphere above). The 1950-control simulations, where these experiments were branched off, are shown in lighter shades. Red line shows observed September sea ice extent from NSIDC.

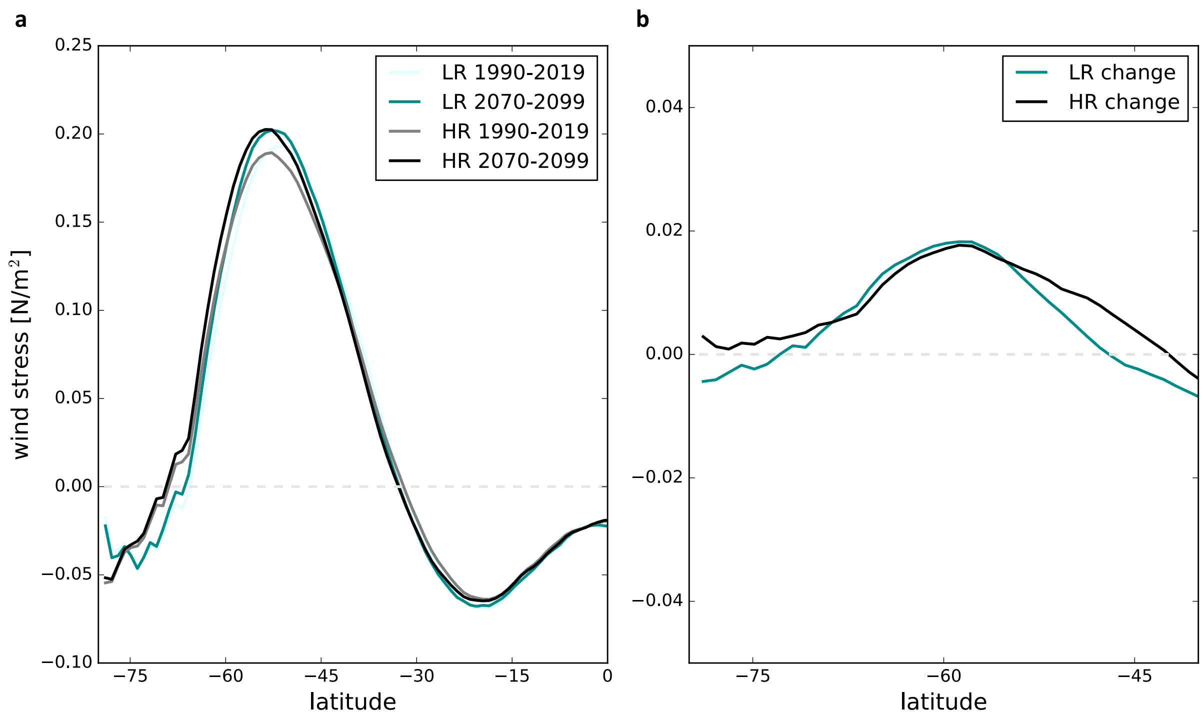

Supplementary Figure 11: Zonal-mean ocean surface wind stress in the Southern Hemisphere. a, When compared to the beginning (1990—2019) of the 21st century, peak wind stress increases towards the end of the century (2070—2099) by about 7% and 5% in the HR and LR scenario simulations, respectively. **b,** Zoom (40°S—80°S) into the changes of wind stress between 1990—2019 and 2070—2099.

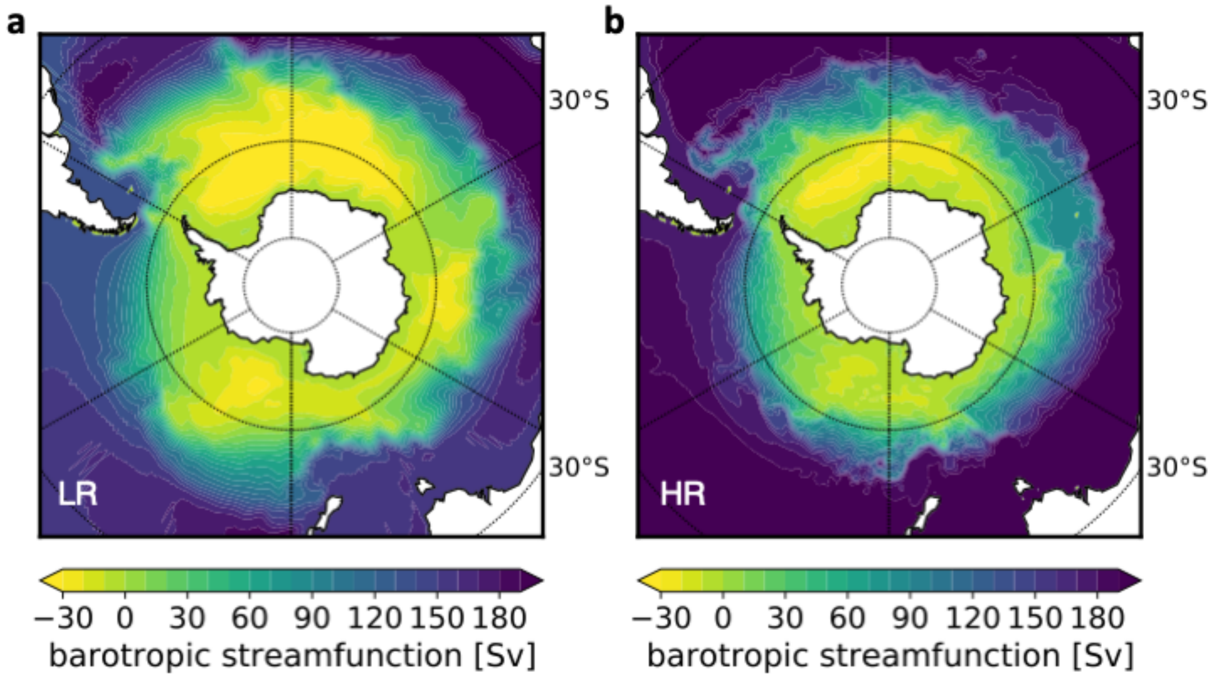

Supplementary Figure 12: Barotropic streamfunction in the Southern Ocean for the different ocean configurations in the 1950-control simulations. a, AWI-CM-LR, **b**, AWI-CM-HR. The ACC transport through Drake Passage (value at Cape Horn) is 133 Sv (LR) and 178 Sv (HR), respectively. To calculate the barotropic streamfunction, the barotropic flow is integrated northwards starting from the Antarctic coast.

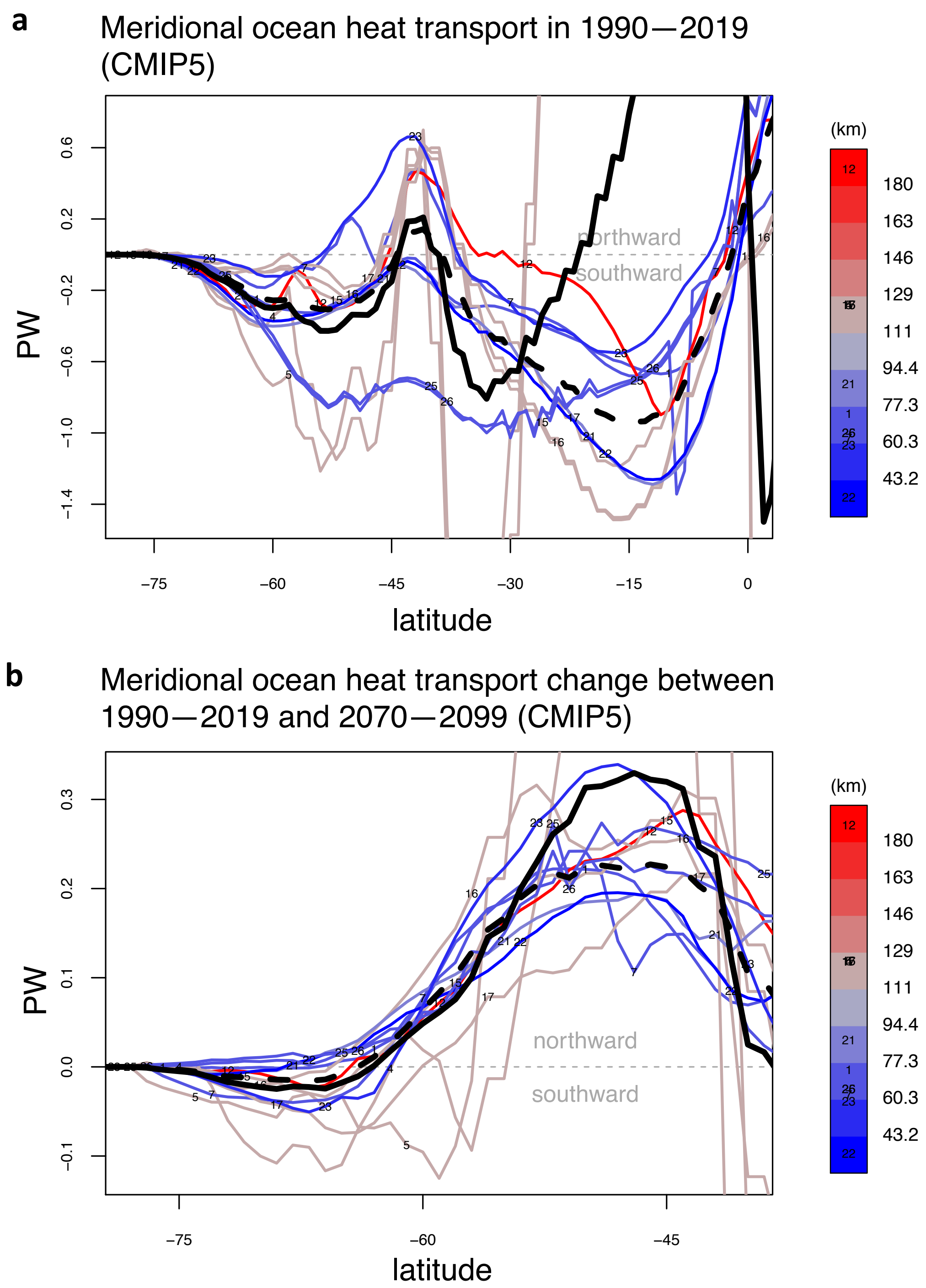

Supplementary Figure 13: Meridional ocean heat transport in the individual CMIP5 models in Petawatt [PW]. a, mean for 1990-2019 and **b,** its climate change signal (2070-2099 minus 1990-2019). The lines are colored according to the average spatial resolution in the Southern Ocean; label numbers represent the individual models as in Fig. 1. Solid black line is the CMIP5 ensemble-mean. Dashed black line is the reduced CMIP5 ensemble-mean without the CMCC model family (models #4 and #5),

which is an outlier northward of approximately 30°S that impacts the ensemble-mean (panel **a**).

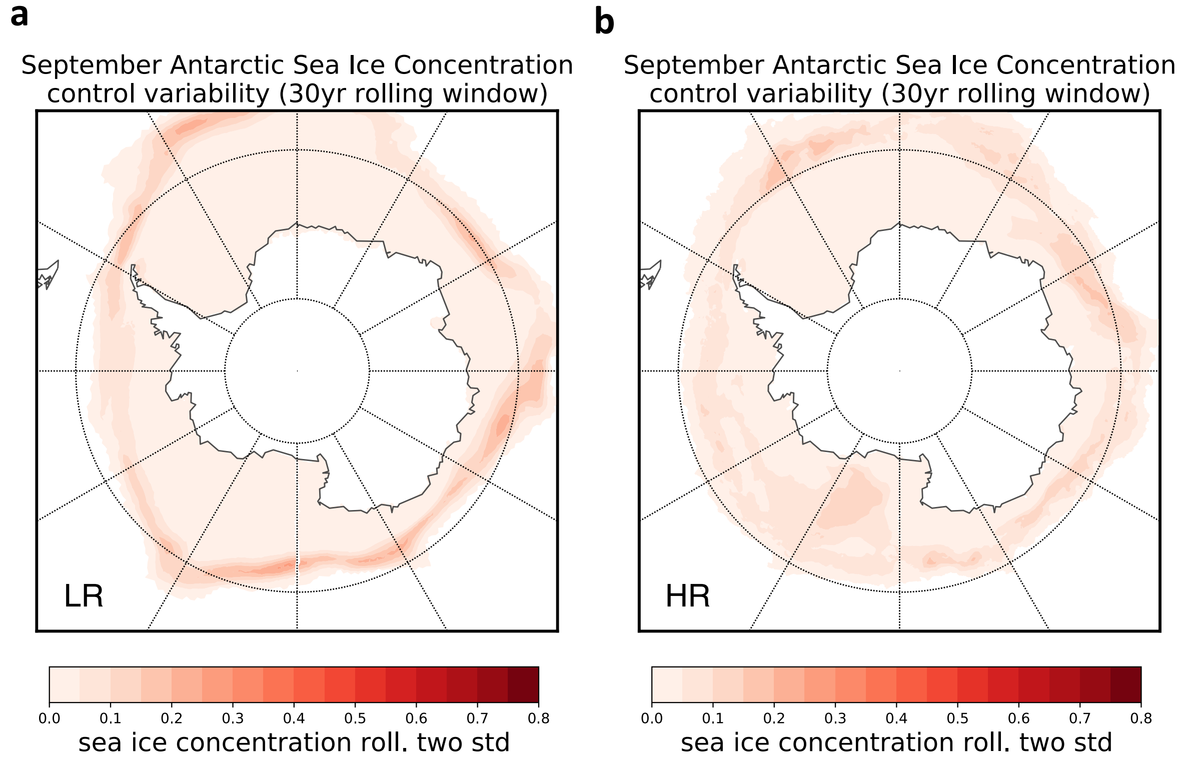

Supplementary Figure 14: Estimates for Antarctic sea ice internal variability from the HR and LR 1950 control simulations. a, Two standard deviations of rolling 30yr-mean Antarctic sea ice concentration in the LR control, and **b,** the same for the HR control. These patterns are used in Fig.3c to test for physical significance of the simulated sea ice concentration response between the two 30yr-periods 2070—2099 (at the end of the 21st century) and 1990—2019 (at the beginning of the 21st century). The colorbar range for the internal variability estimate goes from 0 to 0.8 in order to mimic the sea ice concentration response [-0.8, 0.8] in Fig.3c.

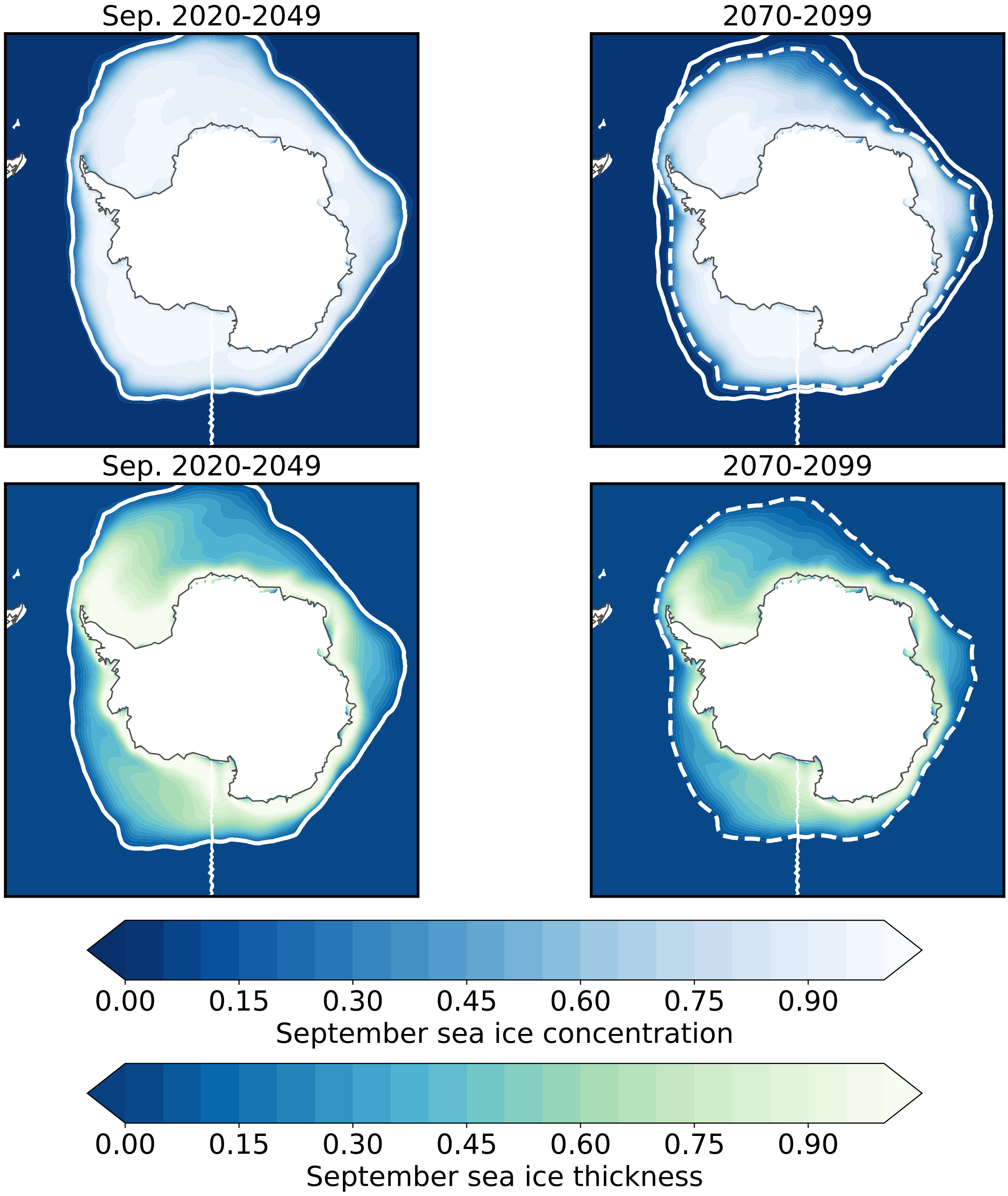

Supplementary Figure 15: LR mean states of ice extent and thickness. 30yr-mean sea ice concentration and thickness [m] for (left column) 2020-2049 and for (right column) 2070-2099 in the LR simulation. White solid contour is sea ice extent (SIC>15%) in 2020-2049. The dashed contours indicate sea ice extent for 2070-2099.

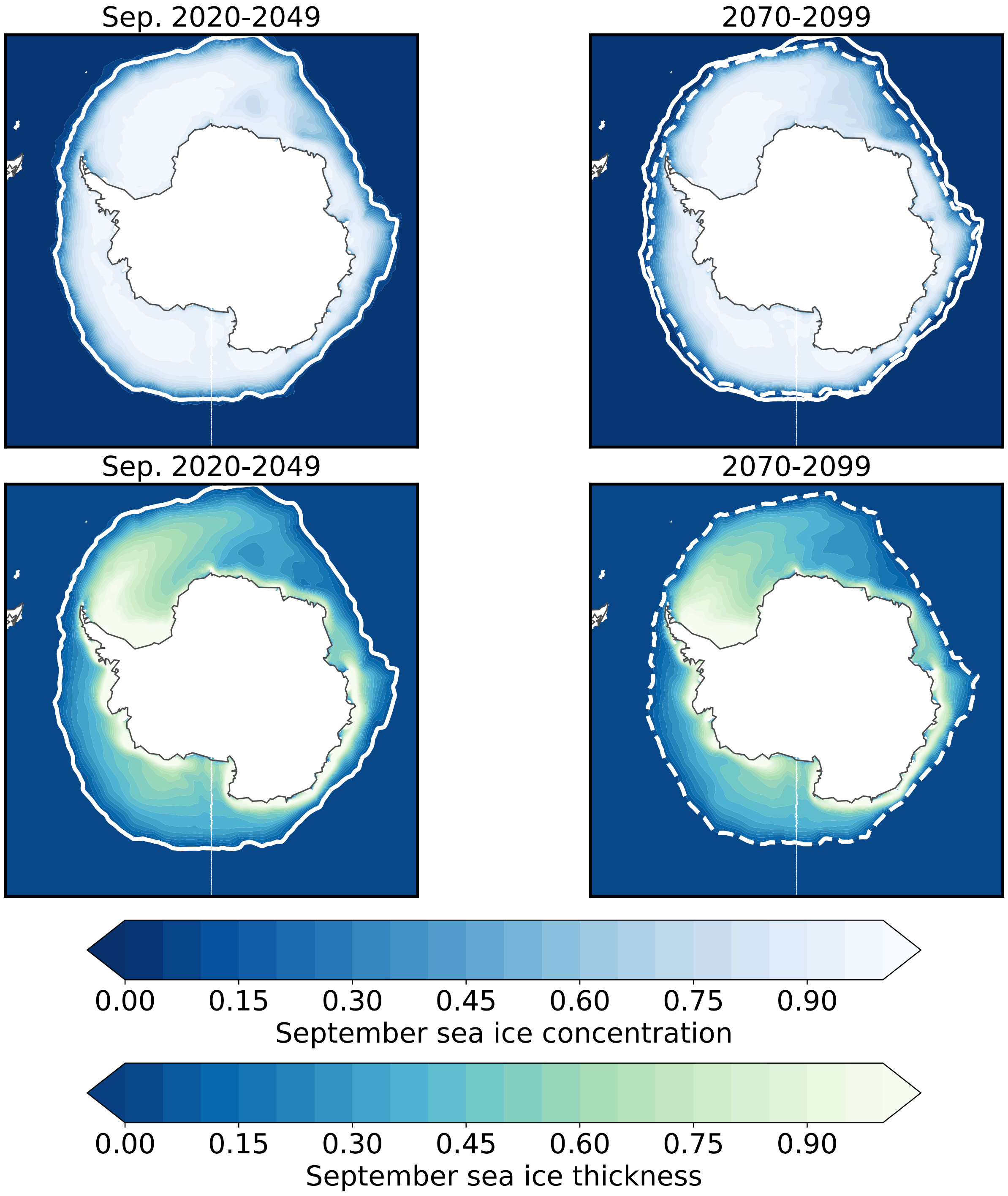

Supplementary Figure 16: HR mean states of ice extent and thickness. 30yr-mean sea ice fraction and thickness [m] for (left column) 2020-2049 and for (right column) 2070-2099 in the HR simulation. White solid contour is sea ice extent (SIC>15%) in 2020-2049. The dashed contours indicate sea ice extent for 2070-2099.

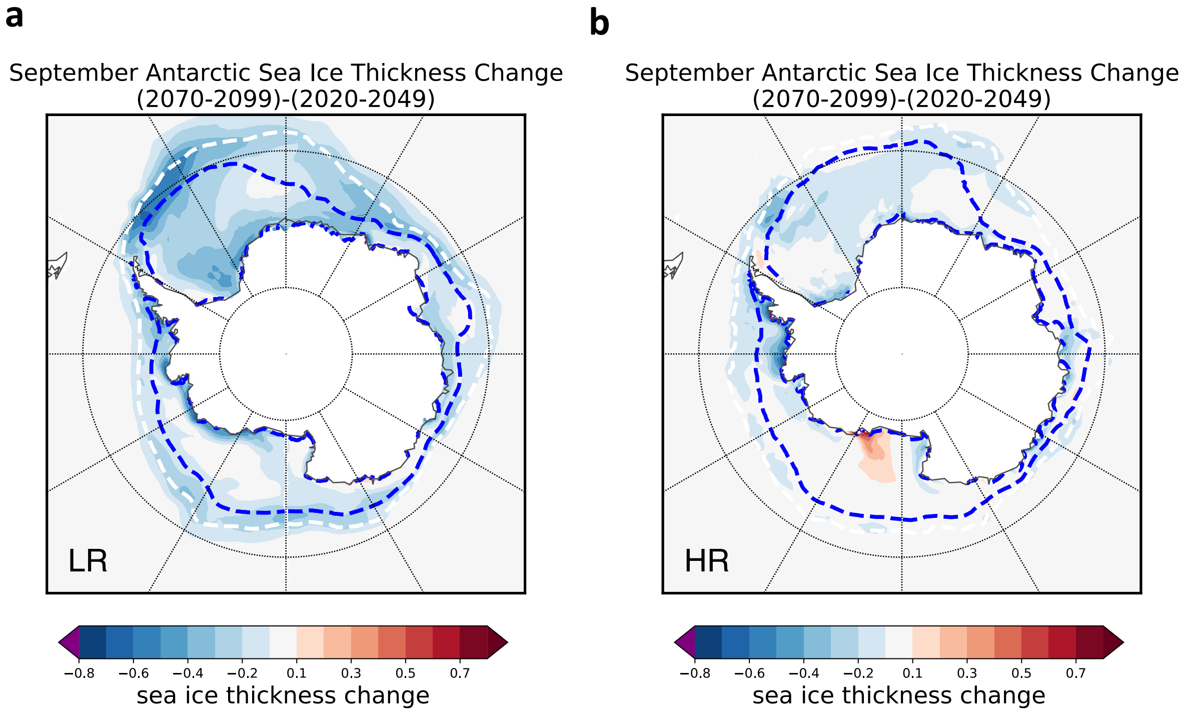

Supplementary Figure 17: September Antarctic Sea Ice Thickness Change (2070-2099 minus 2020-2049). a, thickness change in the LR and **b,** in the HR simulations. Although the 2020—2049 mean state is similar in LR and HR for both thickness and concentration in terms of pattern and magnitude (Supplementary Figures S15 and S16), the thickness change patterns are very different for LR and HR. White dashed contour denotes 15% sea ice concentration contour in 2070—2099; blue dashed is for the 75% sea ice concentration in 2070—2099.
